# Longitudinal Changes of Cognition and Frailty With All-Cause and Cause-Specific Mortality in Chinese Older Adults: An 11-Year Cohort Study

**DOI:** 10.1093/geroni/igad114

**Published:** 2023-10-17

**Authors:** Chen Chen, Xinwei Li, Jun Wang, Jinhui Zhou, Yuan Wei, Yufei Luo, Lanjing Xu, Zuyun Liu, Yuebin Lv, Xiaoming Shi

**Affiliations:** China CDC Key Laboratory of Environment and Population Health, National Institute of Environmental and Health, Chinese Center for Disease Control and Prevention, Beijing, China; China CDC Key Laboratory of Environment and Population Health, National Institute of Environmental and Health, Chinese Center for Disease Control and Prevention, Beijing, China; Department of Epidemiology and Biostatistics, School of Public Health, Jilin University, Changchun, Jilin, China; China CDC Key Laboratory of Environment and Population Health, National Institute of Environmental and Health, Chinese Center for Disease Control and Prevention, Beijing, China; China CDC Key Laboratory of Environment and Population Health, National Institute of Environmental and Health, Chinese Center for Disease Control and Prevention, Beijing, China; China CDC Key Laboratory of Environment and Population Health, National Institute of Environmental and Health, Chinese Center for Disease Control and Prevention, Beijing, China; China CDC Key Laboratory of Environment and Population Health, National Institute of Environmental and Health, Chinese Center for Disease Control and Prevention, Beijing, China; School of Public Health, Anhui Medical University, Hefei, Anhui, China; China CDC Key Laboratory of Environment and Population Health, National Institute of Environmental and Health, Chinese Center for Disease Control and Prevention, Beijing, China; Department of Public Health, Zhejiang University School of Medicine, Hangzhou, Zhejiang, China; School of Public Health and the Second Affiliated Hospital, Zhejiang University School of Medicine, The Key Laboratory of Intelligent Preventive Medicine of Zhejiang Province, Hangzhou, Zhejiang, China; China CDC Key Laboratory of Environment and Population Health, National Institute of Environmental and Health, Chinese Center for Disease Control and Prevention, Beijing, China; China CDC Key Laboratory of Environment and Population Health, National Institute of Environmental and Health, Chinese Center for Disease Control and Prevention, Beijing, China; Center for Global Health, School of Public Health, Nanjing Medical University, Nanjing, Jiangsu, China

**Keywords:** Cardiovascular disease, Cognition, Mortality, Physical frailty, Trajectory

## Abstract

**Background and Objectives:**

Physical function deterioration is always accompanied by a cognitive decline in older adults. However, evidence is lacking for the long-term simultaneous changing patterns of cognition and physical frailty and their associations with mortality among older adults.

**Research Design and Methods:**

This study included 8,231 adults aged ≥65 with a baseline and at least one follow-up assessment of both cognition and physical frailty from the 2007–2018 Chinese Longitudinal Healthy Longevity Survey. Physical frailty (FRAIL phenotype) and cognition (Mini-Mental State Examination) were applied. Group-based joint trajectory modeling was used to fit the joint trajectories of cognition and physical frailty. Cox proportional hazards model was used to evaluate the trajectory-mortality associations.

**Results:**

Three distinct joint trajectories were identified: *no joint progression* (34.4%), *moderate joint progression* (47.0%), and *rapid joint progression* (18.6%). During a median follow-up of 8.3 years, the *rapid joint progression* group, compared to the *no joint progression*, had the highest risk for all-cause mortality (hazard ratio (HR), 3.37 [95% CI: 2.99–3.81]), cardiovascular (CVD) mortality (3.21 [2.08–4.96]) and non-CVD mortality (2.99 [2.28–3.92]), respectively. Joint trajectory was found to be more predictive of mortality as compared to baseline measures of cognition and/or frailty (*C*-statistic ranged from 0.774 to 0.798). Higher changing rates of cognition and frailty were observed among all-cause decedents compared to CVD and non-CVD decedents over a 45-year span (aged 65–110) before death.

**Discussion and Implications:**

Our study suggested that subjects with the worst cognitive decline and severest physical frailty progression were at the highest risk for all-cause and cause-specific mortality. Our findings expand the limited prior knowledge on the dynamic course of cognition and frailty.

**Translational Significance:** Little is known about the long-term simultaneous changing patterns of cognition and physical frailty and their associations with mortality among older adults. We identified those with the worst baseline performance and simultaneous worsening status of cognition and frailty were at the highest mortality risk. Faster-changing patterns were observed among cardiovascular disease decedents over a 45-year span. Our findings highlight the importance of maintaining slower declining rate of cognition and physical function to achieve healthy aging during late-year life. Identifying crucial factors affecting long-term concurrent changes in mental and physical integrity will be useful for distinguishing potential targets of intervention.

## Background and Objectives

Cognitive impairment and physical frailty, two of the most common geriatric conditions, have resulted in substantial burdens on economic, medical, and social costs among older adults worldwide (Panza et al., 2018). The prevalence of cognitive impairment may vary between 9.7% and 23.3% among Chinese adults aged 60 years or older. This means that approximately 39 million older Chinese adults have cognitive impairment (Jia et al., 2020). Cognitive impairment is a transitional stage from unimpaired cognition to dementia when primary interventions should be focused on for preventing dementia. Frailty is a syndrome characterized by diminished physiological reserve and function across multiple organ systems (Clegg et al., 2013). The pooled prevalence of frailty is reported to be 9.9% among Chinese older adults (≥60 years; Ma et al., 2018). In contrast, the estimated population prevalence in previous research is broad, from 2% to 54%, due to the different operational instruments used to define frailty, such as the physical frailty or frailty index. Cognitive frailty is a clinical status of concurrent cognitive impairment and physical frailty among those without a diagnosis of dementia (Kelaiditi et al., 2013), which is proposed based on close associations and potential interactions between these two aging indicators and has gained more attention in recent years. Integrated cognition and physical functions are crucial for older adults to maintain good health and achieve healthy aging.

Clinical research reaches the consensus that frailty process appears to be a dynamic state from robustness to functional decline, which indicates frailty is potentially preventable or reversible (Puts et al., 2017; Solfrizzi et al., 2017). Although cognition and physical frailty generally declines with increased aging, dynamics, and heterogeneity have been observed in the procession of mobility function, cognition, frailty, and aging among older adults (Lowsky et al., 2014; Montero-Odasso et al., 2009; Yang & Lee, 2010). These variations can be measured using joint trajectory model, which could help to clarify the natural course of cognitive frailty and provide a more precise measurement on assessing the simultaneous changes of cognition and physical frailty over time.

It has been confirmed that the use of baseline cognition and frailty (Esteban-Cornejo et al., 2019; Lee et al., 2018; Liu, Chen, et al., 2018; Solfrizzi et al., 2017), that is, measures at a one-time point, allows for predicting mortality. Recently, researchers have been exploring the patterns and shapes of frailty changes throughout later life to identify specific risk factors that can accelerate the rate of frailty changes among older adults followed up for 15 years (Welstead et al., 2021). However, sparse evidence exists regarding the associations of longitudinal changes in frailty with mortality (Bai et al., 2021; Buchman et al., 2009; Chamberlain et al., 2016; Stolz et al., 2021; Stow et al., 2018), especially for cause-specific mortality (Lohman et al., 2020; Taniguchi et al., 2020). As far as we know, only one relevant longitudinal study has been conducted to construct the joint trajectories of cognition and physical frailty among 754 community-living Americans aged ≥70 (Liu, Han, et al., 2018). The study confirmed that the subgroup of cognitive frailty has the highest burden of adverse health outcomes, such as nursing home admission and disability. However, it did not evaluate the associations of joint trajectories with mortality. Therefore, identifying the changing patterns of cognitive frailty evolving along with time and their impacts on mortality in Chinese older adults is urgently needed.

Herein, based on the Chinese Longitudinal Healthy Longevity Survey (CLHLS), a prospective cohort of community-based older adults with four waves of assessments of cognition and physical frailty from 2007 to 2018, we intended to: (1) construct the fitted joint trajectories of cognition and physical frailty among Chinese older adults to explore the simultaneous changing patterns of cognition and frailty over time, (2) evaluate their associations with the risk of all-cause and cause-specific mortality, and (3) delineate the progression patterns and changing rates of cognition and physical frailty over 45 years (aged 65–110) before death among the different types of decedents.

## Research Design and Methods

### Data Sources and Participants

The CLHLS is a community-based prospective cohort study that was initiated in 1998 and conducted in 22 of 31 provinces in China. The follow-up surveys were launched in 2000, 2002, 2005, 2007, 2011, 2014, and 2018. Because of the high mortality rate in older adults, approximately one-third of the subjects were newly recruited from follow-up surveys to achieve a stable sample size for the dynamic cohort. The survey collected comprehensive data, including demographic characteristics, socioeconomic status, lifestyle, and anthropometric measurements. More details of this survey have been described before (Zeng, 2012). The study was approved by the relevant Institutional Ethics Review Board. Written consent was obtained from all participants or their legal representatives.

In brief, 16,954 participants with both baseline cognition and physical frailty assessments were identified from the fifth wave (2007) of CLHLS. We excluded 132 participants aged younger than 65 years old, 227 with baseline dementia, and 8,364 with less than one follow-up physical frailty or cognition assessment, leaving 8,231 older adults who had at least one follow-up assessment of cognition and physical frailty from 2011 through 2018 in the final cohort (Supplementary Figure S1).

### Cognition

Cognition was assessed at the baseline interview and each follow-up survey using the modified version of the widely used Mini-Mental State Examination (MMSE) questionnaire (Katzman et al., 1988). A higher score, increasing from 0 to 30, represents better cognition performance. In CLHLS surveys, for those who were unable to answer or complete a question, we did not repeat the question (Lv et al., 2019). A continuous MMSE score was used in the trajectory analysis, and dichotomous cognition status (participants who had MMSE scores <18 while had no formal schooling, or those with an MMSE score <24 while had at least 1 year of formal schooling were defined as having cognitive impairment; otherwise, defined as normal cognition) was used to construct baseline cognitive frailty (detailed definition seen in Supplementary Material).

### Physical Frailty

Physical frailty was defined by the self-reported FRAIL phenotype with five items (i.e., resistance, ambulation, fatigue, illness, and weight loss; Woo et al., 2015). Each item of the FRAIL phenotype was dichotomized into the interval of 0–1 (details in Supplementary Material). The FRAIL phenotype covers various levels of the function and states of human body, which is a relatively accurate and precise measurement of physical frailty. The continuous FRAIL score was used for trajectory analysis, and dichotomous physical frailty (frail: FRAIL score ≥3; nonfrail: FRAIL score between 0 and 2) was used to construct baseline cognitive frailty.

### Assessment of Death

Information regarding survival status and details of the deaths of the participants were collected during the follow-up surveys (from 2011 through 2018). Those who were lost to follow-up or stayed alive until the end of follow-up (September 1, 2018) were defined as censors. Follow-up time was calculated from the date of the baseline interview until either death or censor, whichever came first. A total of 1,135 of 4,096 deceased participants (27.7%) had documented causes of death. According to the 10th version of the International Classification of Disease (ICD–10), we divided these participants into two groups: cardiovascular disease (CVD) death (ICD–10 codes I00–I99) and non-CVD death (other codes).

### Covariates

Demographics (e.g., age, sex, ethnicity, marital, and education status), socioeconomic (e.g., occupational, residence, living arrangement, and income levels), lifestyle (e.g., current smoking, current alcohol drinking, and regular exercise), social support/factors (e.g., economic independence and adequate medical service), and health status (e.g., heart rate, diastolic blood pressure [DBP], systolic blood pressure [SBP]) were considered based on current literature. Detailed definitions are provided in Supplementary Method section.

### Statistical Analysis

To identify the general patterns of simultaneous changes in cognition and physical frailty during 2007–2018, joint trajectory modeling was performed with the PROC TRAJ macro procedure (Jones & Nagin, 2007), which combined the trajectory of cognition and that of physical frailty to obtain the optimal joint trajectory. The performance of the model fit was assessed by the Bayesian information criterion (BIC) and average posterior probability (Mara & Carle, 2021), and the detailed selection criteria are shown in Supplementary Statistical Analyses section. Descriptive statistics (means ± *SD* or percentages) were used to summarize the baseline characteristics among the full sample and groups stratified by the joint trajectories of cognition and physical frailty.

First, the Cox proportional hazards model was used to evaluate the associations between the joint trajectories and risks of all-cause and cause-specific (CVD and non-CVD) mortality, respectively. Hazard ratios (HR) and 95% confidence interval (CI) were obtained. Three models were constructed with adjustments for different sets of covariates. For model 1, we considered age, sex, education, baseline cognition, and baseline frailty scores. In model 2, we further included smoking, drinking, exercise, adequate medical service, SBP, DBP, and heart rate, in addition to model 1. As for model 3, we additionally adjusted for ethnicity, residence, living arrangement, marital status, income levels, economic independence, and occupational status based on model 2. Kaplan-Meier analysis was used to compare the cumulative mortality of the three death groups. The log-rank test was used to assess the significance of the differences in the joint trajectories. Furthermore, as age and sex are strong determinants of exposure and outcomes, we repeated the above Cox analysis (using model 3) with two subgroups: age (< 80, 80–90, and ≥90 years) and sex (male and female). Interaction items (e.g., joint trajectories × age groups) were included to determine whether the associations may differ by certain factors.

Second, we explored the estimated cognition and physical frailty trajectories over 45 years (aged 65 to 110) to observe the progression patterns and changes in cognition and physical frailty rates before death among all-cause, CVD, and non-CVD decedents using linear mixed-effects models. Repeated measures of cognition and physical frailty were modeled separately as outcomes. We constructed the model with two levels, nesting the longitudinal visit measurements (first level) within the participant (second level) to address data correlations within each individual. Participants were regarded as random effects (both the intercepts and slopes of individuals). Age, sex, and education were modeled as fixed effects. As a time-varying variable, age was also considered in quadratic terms. Sex and education were centered for better interpretability of the coefficient estimates among separate cognition and physical frailty models.

Besides, we conducted four sensitivity analyses with consideration of selection bias, evaluation for predictive ability of joint trajectories, and repeatability of results. First, to assess whether our study had a selection bias, we compared the baseline characteristics among those who were included and excluded. Second, to evaluate whether the baseline measures (e.g., baseline cognition, baseline physical frailty, baseline cognitive frailty; see Supplementary Material) or the concurrent changes (e.g., joint trajectories of cognition and physical frailty) could be more predictive for mortality, we calculated the *C*-statistics and integrated discrimination improvement (IDI) along with net reclassification improvement (NRI) in fully adjusted model 3 with alternative measures (Kang et al., 2015; Pencina et al., 2012; details in Supplementary Material). Third, we repeated the analyses using data from CLHLS 2005–2018 to supplement and validate the previous findings and further verify the joint trajectories and their associations with mortality. Fourth, using the full follow-up measures to construct trajectory in some extent would be a post hoc analysis, so we generated a newly joint trajectory among older adults who both had three assessments of cognition and physical frailty from 2007 through 2014, then tested the prediction power of the joint trajectories on the next 4-year survival.

A two-tailed *p* value < .05 was considered statistically significant. All analyses were performed using SAS version 9.4 (SAS Institute, Cary, NC, USA).

## Results

### Participant Characteristics

A total of 8,231 adults aged 65 and older with a baseline and at least one follow-up assessment of both cognition and physical frailty between 2007 and 2018 were included. The mean (*SD*) age was 83.0 (10.8) years, and approximately 55.1% (*n* = 4,518) were women. Most participants were of Han nationality, were rural residents, and had adequate medical services. And a relatively small proportion of participants lived alone, had high incomes, were currently smoking and drinking, and worked for the government (Table 1).

**Table 1.** Baseline Characteristics of Participants Stratified by the Fitted Joint Trajectories of Cognition and Physical Frailty (*N* = 8,231)

Characteristics	Total (*N* = 8,231)	No joint progression (*N* = 2,830)	Moderate joint progression (*N* = 3,869)	Rapid joint progression (*N* = 1,532)	*p* Values
Age, mean ± *SD*, y	82.9 ± 10.8	74.5 ± 7.7	84.9 ± 9.2	93.1 ± 8.1	<.001
Sex, women (%)	4,518 (54.9)	1,087 (38.4)	2,272 (58.7)	1,159 (75.7)	<.001
Han nationality, yes (%)	7,725 (93.9)	2,651 (93.7)	3,630 (93.8)	1,444 (94.3)	.744
Rural residence, yes (%)	5,088 (61.8)	1,657 (58.6)	2,427 (62.7)	1,004 (65.5)	<.001
Living alone, yes (%)	1,353 (16.4)	376 (13.3)	761 (19.7)	216 (14.1)	<.001
More than 1 year of education, yes (%)^a^	3,509 (42.7)	1,859 (65.8)	1,382 (35.8)	268 (17.6)	<.001
Currently married, yes (%)	3,352 (40.7)	1,831 (64.7)	1,311 (33.9)	210 (13.7)	<.001
Income levels, richer, yes (%)^a^	1,125 (13.7)	449 (15.9)	503 (13.0)	173 (11.3)	<.001
Economic independence, yes (%)	2,544 (30.9)	1,540 (54.4)	842 (21.8)	162 (10.6)	<.001
Current smoking, yes (%)	1,659 (20.2)	816 (28.8)	685 (17.7)	158 (10.3)	<.001
Current alcohol drinking, yes (%)	1,608 (19.5)	763 (27.0)	645 (16.7)	200 (13.1)	<.001
Regular exercise, yes (%)	2,725 (33.1)	1,262 (44.6)	1,160 (30.0)	303 (19.8)	<.001
Adequate medical service, yes (%)	7,681 (93.3)	2,715 (95.9)	3,556 (91.9)	1,410 (92.0)	<.001
Occupation, governmental, yes (%)^a^	644 (7.8)	381 (13.5)	211 (5.5)	52 (3.4)	<.001
SBP, mean ± *SD*, mmHg^a^	134.6 ± 24.3	133.5 ± 22.1	135.5 ± 25.3	134.4 ± 25.4	<.001
DBP, mean ± *SD*, mmHg^a^	77.5 ± 13.4	77.8 ± 12.5	77.4 ± 13.8	77.1 ± 14.2	.407
Heart rate, mean ± *SD*, bpm^a^	72.3 ± 11.6	72.1 ± 10.4	72.4 ± 11.5	72.5 ± 14.1	.042
MMSE score, mean ± *SD*	23.7 ± 8.2	28.2 ± 2.5	24.4 ± 6.0	13.7 ± 10.8	<.001
Physical frailty score, mean ± *SD*	1.3 ± 1.2	0.3 ± 0.6	1.6 ± 1.1	2.3 ± 1.1	<.001

*Notes*: SBP = systolic blood pressure; DBP = diastolic blood pressure; MMSE = Mini-Mental State Examination.

^a^Of the 8,231 older adults, numbers of missing data ranged from 13 to 313 (18 for education, 17 for income levels, 13 for occupational status, 309 for SBP and DBP, and 313 for heart rate).

### Joint Trajectories of Cognition and Physical Frailty

**Figure 1.**
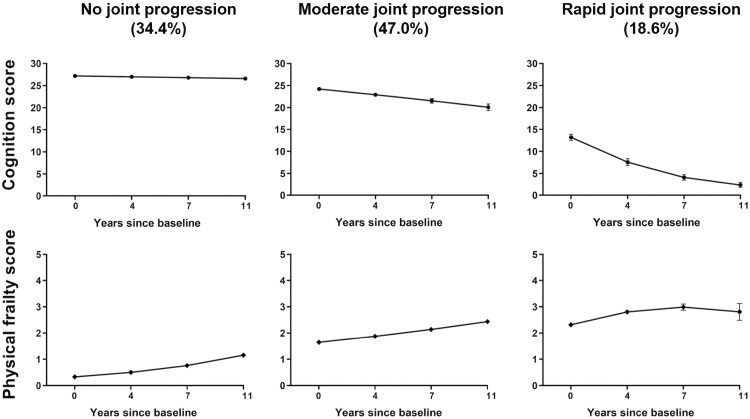
Fitted joint trajectories of cognition and frailty among the CLHLS older adults (2007–2018). Cognition was assessed using the Mini-Mental State Examination (MMSE, score range: 0–30). Higher score indicates better cognition. Physical frailty was defined by the self-reported FRAIL phenotype with five components (fatigue, resistance, ambulation, illness, and weight loss). A higher score indicates worse frailty. ●, ◆ and the error bars represent the predicted values and 95% confidence intervals for MMSE score and physical frailty score, respectively. Three joint trajectories of cognition (top panel) and frailty (bottom panel) were identified as *no joint progression* (*n* = 2,830), *moderate joint progression* (*n* = 3,869), and *rapid joint progression* (*n* = 1,532).

Three distinct trajectories were identified according to baseline cognition and physical frailty scores and their follow-up measurements over time (Figure 1). The *no joint progression* group (predicted group probability: 34.4%) included participants who showed no cognitive impairment (MMSE score <18) and no prefrail (FRAIL scores <2) throughout the 11 years of follow-up. On the contrary, those in the *rapid joint progression* group (18.6%) suffered the worst baseline performance and steeper change rate in both cognition declining and physical frailty increasing over time. About half of older adults fell into *moderate joint progression* group (47.0%), showing progressive physical frailty and slow cognitive decline. Across the three trajectories, the proportions of baseline characteristics differed significantly (all *p < .05) except for Han nationality, DBP, and heart rate (Table 1). The optimal model for the three groups achieved an excellent average posterior probability of assignment, ranging from 0.88 to 0.91 across the three trajectories (Supplementary Table S1). The maximum likelihood estimates and the BIC index were evaluated for the final three groups of joint trajectories, and the results were summarized in Supplementary Table 2.

### Associations of the Joint Trajectories With All-Cause, CVD, and Non-CVD Mortality

#### All-cause mortality

**Figure 2.**
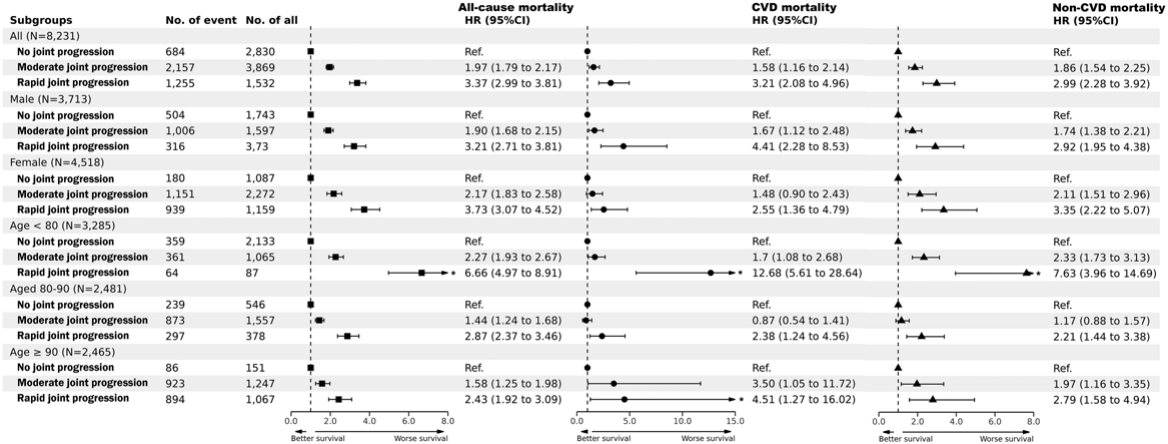
Associations of the joint trajectories with the risk of all-cause, CVD, and non-CVD mortality. Results in all and specified subgroups were shown, stratified by age (<80, ≥80–90, and ≥90 years) and sex (male and female). *Represents that the 95% confidence interval range is too large to display the actual interval in the graph, and the actual range is presented on the right. All models were adjusted for age (not in age subgroup) and sex (not in sex subgroup), education, baseline cognition, baseline frailty, smoking, drinking, exercise, adequate medical service, SBP, DBP, heart rate, ethnicity, residence, living arrangement, marital status, income levels, economic independence, and occupational status. The estimated HRs for each group were compared with *no joint progression* group (reference, HR = 1.0).

**Figure 3.**
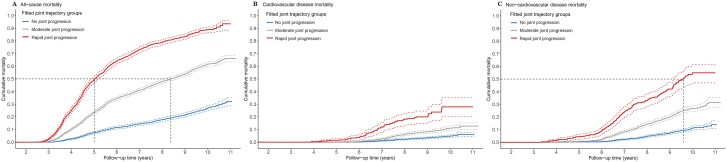
The cumulative incidence of all-cause (A), cardiovascular (B), and noncardiovascular mortality (C) by joint trajectories.

During a median follow-up of 8.3 years (IQR: 5.2–9.6 years), almost half (*n* = 4,096, 49.8%) of the older adults died. We found that worse joint trajectories were associated with higher risk of all-cause mortality. Compared to the *no joint progression* group, participants in the *rapid joint progression* group had a 3.4-fold higher risk of all-cause mortality (adjusted HR, 3.37 [95% CI: 2.99–3.81]) after full adjustment (Supplementary Table 3). Subgroup analyses stratiﬁed by age and sex did not attenuate the risk estimates among all-cause mortality (Figure 2). Joint trajectories of cognition and physical frailty-mortality associations were more pronounced in the younger older adults (<80 years; *p*_trajectories × age groups_ < .01), whereas the associations were not significantly differed by the sex subgroup (all *p*_trajectories × sex_ > .05). The cumulative incidence of all-cause mortality is displayed in Figure 3, that is, worse joint trajectories were significantly associated with higher all-cause mortality (*p*_log-rank_ < .05). For the most favorable (*no joint progression*) and least favorable (*rapid joint progression*) trajectories, the observed rates of all-cause mortality were 2.6% and 25.1% at 4 years, 14.4% and 73.1% at 7 years, and 32.2% and 93.6% at 11 years, respectively (Supplementary Table 4).

#### CVD and non-CVD mortality

During a median follow-up of 9.0 years (IQR: 8.5–10.1 years for CVD and non-CVD deaths), about 6.9% (305/4,440) and 16.7% (830/4,964) older adults died of CVD and non-CVD diseases, respectively. Similar trends of higher mortality risk associated with worse joint trajectories were observed in both CVD and non-CVD deaths (Figure 2). After full adjustment, the *rapid joint progression* group had comparable risk estimates on CVD mortality and non-CVD mortality compared to the *no joint progression* group (model 3, CVD mortality HR 3.21 [95% CI: 2.08–4.96]; non-CVD mortality HR 2.99 [95% CI: 2.28–3.92]). Subgroup analyses considering age and sex did not change the trends of increasing HRs for worse trajectories regarding non-CVD mortality. However, for CVD mortality, the associations became insignificant for the intermediate trajectory (*moderate joint progression*) in both age and sex subgroups, such as in the 80–90-year-old group (adjusted HR _moderate joint progression vs no joint progression_, 0.87 [95% CI: 0.54–1.41]) and in females (adjusted HR _moderate joint progression vs no joint progression_, 1.48 [95% CI: 0.90–2.43]).

Overall, worse joint trajectories were associated with higher cumulative mortality. Each of the three incidence curves was separated after about 6 years of follow-up regarding CVD and non-CVD mortality (*p*_log-rank_ < .05, Figure 3). For the best (*no joint progression*) and worst (*rapid joint progression*) trajectories, observed rates of CVD mortality at 4 years were 0.0 and 0.3%, at 7 years were 1.1% and 11.9%, and at 11 years were 6.0% and 27.9%, respectively. For the best (*no joint progression*) and worst (*rapid joint progression*) trajectories, observed rates of non-CVD mortality at 4 years were 0.0 and 1.4%, at 7 years were 2.3% and 23.9%, and at 11 years were 14.0% and 54.9%, respectively (Supplementary Table 4).

### Age Trajectories of Cognition and Physical Frailty Among Deceased Older Adults With Specific Causes of Death

**Figure 4.**
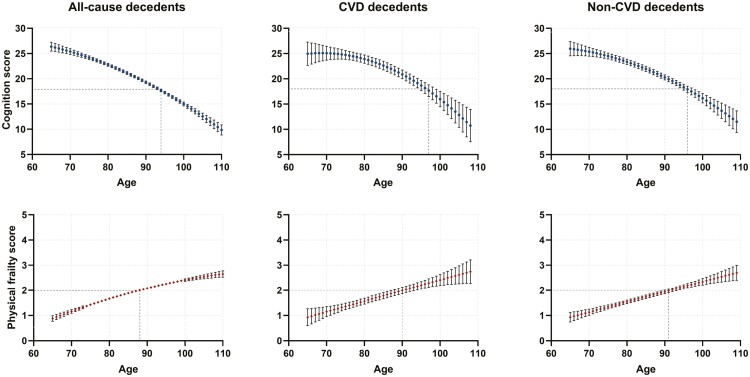
Age trajectories of cognition and frailty throughout about 45 years before death among the decedents. Results for all-cause (left), CVD (middle), and non-CVD (right) were shown. Group symbols are as follows: ●, ◆ represent the predicted values for MMSE score and frailty score at specific age, respectively. The error bars represent 95% confidence intervals for predicted values. Cognitive impairment, defined as with an MMSE score less than 18. Pre-frail, defined as those who met 1 to 2 criteria of the 5 items. The specified ages of occurrence of CI among all-cause, CVD, and non-CVD decedents are 94, 97, and 96 years, respectively; and the corresponding ages of prefrail (FRAIL score = 2) are 88, 90, and 91 years, respectively.

To observe the progression patterns and change rates of cognition and physical frailty among the deceased participants with different causes of death, we compared the estimated cognition and physical frailty trajectories over 45 years (aged 65–110) before the participants died. The most notable thing was that higher changing rates of cognition and physical frailty were observed among all-cause compared to the CVD and non-CVD decedents (Figure 4). Specifically, those in the all-cause mortality panel had the earliest onset ages of cognitive impairment (94 years old) and prefrailty (88 years old); the corresponding age of FRAIL score at 2 were 88 years, 90 years, and 91 years, respectively.

### Sensitivity Analyses

In our sensitivity analyses, we observed the following findings. First, those who were excluded (*n* = 8,723) were older, more likely to be women, and had a higher prevalence of physical frailty and cognitive impairment compared to the included ones (*n* = 8,231). Other comparisons are presented in Supplementary Table 5.

Second, the results of prediction performance on mortality among baseline cognition and frailty predictors and joint trajectory groups are shown in Supplementary Table 6. Joint trajectory was more predictive of mortality as compared to three baseline measures, which had the highest predicted *C*-statistic for all-cause and cause-specific mortality, ranging from 0.774 (95% CI: 0.764–0.785) to 0.798 (95% CI: 0.789–0.808). It showed significant improvements in the IDI and NRI, predicting three mortality types. Compared with baseline cognitive frailty, joint trajectories showed significant improvements in IDI 0.0241 (0.0206, 0.0276) and NRI 0.3940 (0.3508, 0.4372) in predicting all-cause mortality; whereas, *baseline cognition* and *baseline frailty*, compared to *baseline cognitive frailty*, had lower improvements or did not meet statistical significance. Similar results were observed between IDI and NRI in predicting CVD and non-CVD mortality.

Third, using CLHLS data from 2005 through 2018 with 7,452 participants, we confirmed that the trend of worse joint trajectories associated with higher risks of mortality was robust. After full adjustment, the *rapid joint progression* group had comparable risk estimates for all-cause and non-CVD mortality [all-cause mortality HR 3.11 (95% CI: 2.74–3.54), non-CVD mortality HR 3.23 (95% CI: 2.34–4.44)] and substantial improvements in predicting death (Supplementary Tables 7 and 8).

Fourth, among 4,966 older adults who both had three assessments of cognition and physical frailty from 2007 through 2014, two newly generated three-wave joint trajectories were identified (Supplementary Figure 2, Supplementary Tables 9 and 10): *no joint progression* (*n* = 3,925, predicted group probability: 79.0%) and *moderate joint progression* (*n* = 1,041, 21.0%). And the Cox results demonstrated that the three-wave joint trajectory could significantly predict the next 4-year survival (Supplementary Table 11).

## Discussion and Implications

We identified three specific joint trajectories based on 8,231 Chinese adults aged 65 years or older, with a baseline and at least one follow-up assessment of cognition and physical frailty from 2007 through 2018. *Rapid joint progression* (the least favorable subgroup) exhibited the worst baseline performance and steeper change rate on both cognition declining and physical frailty aggravating over time. Worse joint trajectories were significantly associated with higher risks for all-cause, CVD, and non-CVD mortality. The joint trajectory was more predictive of mortality compared to baseline measures. Furthermore, similar progression patterns, whereas higher changing rates of cognition and physical frailty, were observed among all-cause decedents compared to CVD and non-CVD decedents.

The three joint trajectories of cognition and physical frailty identified in our study confirmed the considerable subgroup-based heterogeneity between the changing patterns of cognition and physical frailty declining among older adults. Similarly, a recent study regarding older Mexican Americans aged ≥65 also identified a three-subgroup pattern on longitudinal changes in cognition and frailty during the 18-year follow-up (Howrey et al., 2020). However, it focused on revealing predictors of trajectories instead of evaluating the effects of joint trajectories on adverse health outcomes. Our results showed the *rapid joint progression* group had highest risk for all-cause and non-CVD mortality, which is consistent with prior studies that used baseline cognitive frailty (Esteban-Cornejo et al., 2019; Lee et al., 2018; Liu, Chen, et al., 2018), suggesting that older adults with cognitive frailty represent a vulnerable subgroup with an extremely elevated risk of survival.

The closely simultaneous changes in cognition and physical frailty over time and their joint contributions to the increased mortality risk provide a deeper explanation for the relatively novel construction—cognitive frailty. The concurrent changes between cognition and physical frailty suggest that they might share the same underlying pathophysiologic pathways or mechanisms (Halil et al., 2015; Robertson et al., 2013). Notably, one shortage in our fitted trajectories is that the changes in the intermediate trajectory are not so evident across the 11-year follow-up. Therefore, we did not find an exact time point at which the cognition and physical frailty status transitioned from normal to meet the criteria for cognitive frailty. Nevertheless, Liu, Han, et al. (2018) found that at about 36 months from baseline , participants in the intermediate group experienced cognitive impairment and frailty status, as well as in the cognitive frailty group. Therefore, more prospective cohorts of Chinese older adults with shorter intervals and more frequent follow-ups are needed to verify whether information bias exists in the trajectories we constructed in this study.

In the current study, several possible potential speculations may elucidate the observed higher change rates of cognition and physical frailty among all-cause decedents compared to CVD and non-CVD decedents. First, our results (Supplementary Table 6) per se demonstrated that concurrent changing of cognition and frailty exhibited better prediction performance on all-cause mortality compared to CVD and non-CVD mortality after full adjustment (*C*-statistic point estimates were 0.798, 0.774, and 0.790 for all-cause, CVD, and non-CVD mortality, respectively). This result suggests that concurrent changing of cognition and frailty may have high discriminating power and sensitivity to all-cause death. Second, some gerontologists suggest that cognitive frailty is a manner of preclinical cognitive status caused by physical frailty instead of neurodegenerative disorders (Arai et al., 2018; Xue et al., 2019). The decline in physical function contributes to both the etiology and outcome of the pathological state of cognitive frailty. Therefore, joint trajectories could capture relatively fewer CVD-related adverse health outcomes, as cognitive problems are more closely associated with CVD disease. Third, the relatively small sample size among CVD and non-CVD decedents, and the few older adults having three or more measurements of cognition and physical frailty to construct the joint trajectory, might cause an underestimation of the changing rate in cognition and physical frailty among these two groups of decedents.

The strengths of this study include the prospective cohort design, a nationally representative sample population with a considerably large size, and the multiwave face-to-face assessments over 11 years, which all provided us with a unique opportunity to explore the simultaneous changing patterns and rates of cognition and frailty over time among Chinese older adults. We also tried to evaluate the joint trajectories of cognition and physical frailty and their associations with mortality by repeating analyses in the participants from wave 2005 to 2018. The comparable results helped verify our conclusions. Furthermore, our results demonstrated that joint trajectory was more predictive of mortality than baseline measures, which substantially underestimated the actual change rate of frailty in the longitudinal models.

However, several limitations should be considered. First, we acknowledge that the high attrition rate and potential survival bias might attenuate the validity of our results. We demanded at least two waves of measurements for cognition and physical frailty to construct the joint trajectories. Thus, approximately half of the older adults were excluded from our analyses. Yet, as this disadvantage seems inevitable for considerable epidemiological research, the trajectory modeling method we used is flexible and can handle randomly missing data. Second, since no previous gait speed and grip strength measurements were collected, we could not construct the Fried physical frailty, though it is more widely used and easier to compare with other studies. Nevertheless, in our study, we aimed to describe the heterogeneity of baseline cognition and physical frailty and their changes over time, where the FRAIL phenotype could also reflect various levels of the functional states and is a relatively accurate and precise measurement of physical frailty. Third, although our study provides evidence of the association between longitudinal changes in cognition and frailty and cause-specific mortality, the relatively small sample size might cause an underestimation of the risk effects. Fourth, the covariates included in our models were relatively limited with health-related assessment regarding disability or extensive chronic diseases absent. Future study may consider comprehensive adjustment to further improve the accuracy and robustness.

By exploring the concurrent change patterns of cognition and physical frailty over time, we identified three distinct joint trajectories among Chinese older adults. The results confirmed that subjects in the *rapid joint progression* group were at the highest risk for both all-cause and cause-specific mortality. With our findings, we expanded the limited prior knowledge on the natural dynamic progression course of cognition and frailty, which could provide more insights into public health management and clinical care for this highly vulnerable subgroup of older adults.

## Supplementary Material

igad114_suppl_Supplementary_Figures_S1-S2_Tables_S1-S11
