# Supplementary material for: Longitudinal Changes of Cognition and Frailty With All-Cause and Cause-Specific Mortality in Chinese Older Adults: An 11-Year Cohort Study
Source: Innov Aging. 2023 Oct 17;7(9):igad114. doi: 10.1093/geroni/igad114 (PMC10681360; doi:10.1093/geroni/igad114)
Supplement: igad114_suppl_Supplementary_Figures_S1-S2_Tables_S1-S11 [file igad114_suppl_supplementary_figures_s1-s2_tables_s1-s11.docx]

**Online Supplementary Material**

**Contents**

[**Methods** 35](#_Toc136982566)

[**Cognition** 35](#_Toc136982567)

[**Physical Frailty** 35](#_Toc136982568)

[**Baseline Cognitive Frailty** 35](#_Toc136982569)

[**Assessment of Death** 35](#_Toc136982570)

[**Covariates** 35](#_Toc136982571)

[**Statistical Analysis** 36](#_Toc136982572)

[**Supplementary References** 38](#_Toc136982573)

[**Figure S1. Flow chart of participants enrollment and analyses procedures of this study** 39](#_Toc136982574)

[**Figure S2. Fitted joint trajectories among the CLHLS older adults with both three assessments of cognition and physical frailty (year 2007 – 2014, N=4,966).** 40](#_Toc136982575)

[**Table S1. Posterior Probability of Assignment (PPA) of the 3 groups according to Joint Trajectory Model** 41](#_Toc136982576)

[**Table S2. Estimated joint trajectories and group-specific growth parameters** 42](#_Toc136982577)

[**Table S3. Hazard ratios (95% CI) for all-cause, CVD, and non-CVD mortality according to the fitted joint trajectories of cognition and physical frailty for participants enrolled between 2007 and 2018** 43](#_Toc136982578)

[**Table S4. Cumulative incidences for all-cause death, CVD death, and non-CVD death in the Chinese older adults across 11 years of follow-up according to the fitted joint trajectories of cognition and physical frailty [Wave 2007-2018]** 44](#_Toc136982579)

[**Table S5. Baseline characteristics of the included vs excluded participants (N=16954) [Wave 2007-2018]** 45](#_Toc136982580)

[**Table S6. Prediction performance on mortality among baseline cognition and / or frailty predictors and the joint trajectories using CLHLS data [Wave 2007-2018]** 46](#_Toc136982581)

[**Table S7. Hazard ratios (95% CI) for all-cause, CVD, and non-CVD mortality according to the fitted joint trajectories of cognition and physical frailty [Wave 2005-2018]** 47](#_Toc136982582)

[**Table S8. Prediction performance on mortality among baseline cognition and frailty predictors and the joint trajectories [Wave 2005-2018]** 48](#_Toc136982583)

[**Table S9. Posterior Probability of Assignment (PPA) of the 2 groups according to Joint Trajectory Model among older adults who had both three assessments of cognition and physical frailty (Wave 2007 – 2014, N=4,966)** 49](#_Toc136982584)

[**Table S10. Estimated joint trajectories and group-specific growth parameters among older adults who had both three assessments of cognition and physical frailty (Wave 2007 – 2014, N=4,966)** 50](#_Toc136982585)

[**Table S11. Hazard ratios (95% CI) for all-cause, CVD, and non-CVD mortality according to the fitted joint trajectories among older adults who had both three assessments of cognition and physical frailty (Wave 2007 – 2014, N=4,966)** 51](#_Toc136982586)

**Methods**

**Cognition**

Six domains (i.e., concentration, memory recall, language, orientation, working memory, and visuospatial ability) were included with a maximum score of 30.

**Physical Frailty**

*Resistance* was met if participants could not continuously climb 10 steps without rest and assistance. *Ambulation* was met if the participants could not walk 1 km continuously at a time by themselves. *Fatigue* was determined if participants answered “frequently” or “always” for either of the two questions from the Center for Epidemiological Studies Depression scale (CES-D): “I could not keep up with my current state of life” and “I feel like I'm struggling to do anything”. *Illness* status was met if participants self-reported having more than 5 of chronic conditions, i.e., hypertension; diabetes or hyperglycemia; cancer; chronic lung disease; heart problems; stroke; kidney disease; stomach or other digestive disease; arthritis or rheumatism; and asthma. *Loss of weight* was defined as self-reported loss of 5 or more kilograms in the last year or body mass index (BMI) ≤ 18.5 kg/m^2^.

Among the 5 indicators of physical frailty, fatigue and weight loss represent the biological function, resistance and ambulation represent the physical function, and illness represents the multi-system and multi-factor accumulation status. Each indicator represents one score for frailty assessment.

**Baseline Cognitive Frailty**

Baseline cognitive impairment was defined as with an MMSE score < 18 while had no formal schooling, or with an MMSE score < 24 while had at least 1 year of formal schooling; otherwise, defined as having normal cognition. Participants who met three or more criteria were defined as having physical frailty (frail, ≥ 3); otherwise, they were considered as having no physical frailty (nonfrail, 0 - 2). In line with the definition by an (I.A.N.A./I.A.G.G.) international consensus group (Kelaiditi et al., 2013), *cognitive frailty* was defined as the simultaneous presence of both cognitive impairment and physical frailty and has been previously validated (Chen et al., 2020; Chu et al., 2019; Shimada et al., 2018). Based on the two baseline components—cognitive impairment and physical frailty, we defined four combined groups:

Group 1: normal cognition and non-frail status

Group 2: cognitive impairment and non-frail status

Group 3: normal cognition and frail status

Group 4: cognitive impairment and frail (cognitive frailty)

Continuous form of baseline cognitive frailty was used to model performance index, such as C-statistic, integrated discrimination improvement (IDI), and net reclassification improvement (NRI).

**Assessment of Death**

Exact death dates were collected from their closest family members or verified by qualified doctors. Information regarding cause-specific mortality was collected from the relatives of deceased individuals through face-to-face interviews for each wave or through an additional interview via phone call in the 2014 survey, which was subsequently assessed by qualified doctors in the 2018 survey.

**Covariates**

Demographics (e.g., age, sex, ethnicity, marital, and education status), socioeconomic (e.g., occupational, residence, living arrangement, and income levels), lifestyle (e.g., current smoking, current alcohol drinking, and regular exercise), social support/factors (e.g., economic independence and adequate medical service), and health status [e.g., heart rate, diastolic blood pressure (DBP), systolic blood pressure (SBP)] were considered based on current literature.

Education was categorized as having more than 1 year of schooling or no formal education. Residence was divided as urban (city residence) or rural (town or countryside residence). Income levels was classified as “richer” or “general or lower” according to the answers of participants to the question “Compared with other locals, how do you think about your economic position?” Current smoking and alcohol drinking were categorized as current smoker/drinker or no present smoking/drinking behavior status. Regular exercise (yes vs. no) was determined according to the answers of participants as “almost every day” or “at least once per week” for the question “Do you do exercises regularly at present, including walking, playing ball, running, and Qigong?”

**Statistical Analysis**

**Joint Trajectory Model**

In this group-based trajectory model, all subjects were divided into several groups as they had heterogeneous features between groups and homogeneous features within each group. The models were developed with distinct functional (e.g., intercept only, linear, quadratic, and cubic) and different order terms. Continuous MMSE score (range: 0 - 30) was modeled with censored normal model. Continuous count of frailty measures (range: 0 - 5) was modeled as zero-inﬂated Poisson distribution. Changes in cognition and frailty was modeled as a function of the actual follow-up time (years, from baseline to each cognition or frailty assessment) rather than the current age, as we were here interested in exploring the general patterns of simultaneous changes in cognition and frailty during the 11 years (2007 – 2018), but not to determine the exact age of occurrence with any outcomes.

Model fit was assessed using: (1) Bayesian Information Criteria (BIC, lower absolute value is generally better), and comparisons of BIC values between alternative models. (2) The average posterior probability (AvePP) of assignment (a probability of assignment ≥ 0.9 was considered an excellent ﬁt and a value < 0.7 was considered a poor ﬁt), proportion of group membership with a posterior probability of assignment < 0.7, and the differences between the predicted group probability and observed group proportions (Nagin, 1999). The final model for cognition and frailty both included one quadratic trajectory and two linear trajectories. The joint trajectories combined the cognition and frailty trajectory, which also provided the predicted group probabilities of frailty group membership given membership in a cognition trajectory.

**Linear Mixed-effects Model**

In this step, we transformed the long format dataset to wide format, and each participant could contribute multiple observations. Proc PLM procedure was used to fit predicted values of cognition and frailty aged from 65 - 110. Repeated measures of cognition and physical frailty were modeled separately as outcomes. We constructed the model with two levels, nesting the longitudinal visit measurements (first level) within the participant (second level) to address data correlations within each individual. Participants were regarded as random effects (both the intercepts and slopes of individuals). Age, sex, and education were modeled as fixed effects. As a time-varying variable, age was also considered in quadratic terms. Sex and education were centered for better interpretability of the coefficient estimates among separate cognition and physical frailty models.

**Predictive Performance of Joint Trajectories for Mortality**

We calculated the C-statistic, IDI, and NRI (Pencina et al., 2008) in comparison of joint trajectories of cognition and frailty to those of the baseline measures (such as baseline cognitive frailty). The C-statistic measured the consistency between model estimates and observed events. NRI and IDI were calculated as the added predictive values between models using *joint trajectories* with that using *baseline cognitive frailty*, as well as the added predictive values based on *baseline cognition* or *baseline frailty* vs. *baseline cognitive frailty*. If the IDI > 0, there is a positive improvement, indicating that the new model has improved predictive power than the old model, while an IDI < 0 represents a negative improvement with decreased predictive power of the new model. And if the IDI = 0, the new model is considered to have no improvement. The NRI has similar criteria with the IDI values. With a given cut-off, NRI might be a better choice; otherwise, IDI may be preferred.

**Supplementary References**

Chen, C., Park, J., Wu, C., Xue, Q., Agogo, G., Han, L., Hoogendijk, E., Liu, Z., & Wu, Z. (2020). Cognitive frailty in relation to adverse health outcomes independent of multimorbidity: Results from the China health and retirement longitudinal study. *AGING-US*, *12*(22), 23129–23145.

Chu, N., Bandeen-Roche, K., Tian, J., Kasper, J., Gross, A., Carlson, M., & Xue, Q. (2019). Hierarchical Development of Frailty and Cognitive Impairment: Clues Into Etiological Pathways. *JOURNALS OF GERONTOLOGY SERIES A-BIOLOGICAL SCIENCES AND MEDICAL SCIENCES*, *74*(11), 1761–1770. https://doi.org/10.1093/gerona/glz134

Kelaiditi, E., Cesari, M., Canevelli, M., van Kan, G. A., Ousset, P.-J., Gillette-Guyonnet, S., Ritz, P., Duveau, F., Soto, M. E., Provencher, V., Nourhashemi, F., Salvà, A., Robert, P., Andrieu, S., Rolland, Y., Touchon, J., Fitten, J. L., & Vellas, B. (2013). Cognitive frailty: Rational and definition from an (I.A.N.A./I.A.G.G.) international consensus group. *The Journal of Nutrition, Health & Aging*, *17*(9), 726–734. https://doi.org/10.1007/s12603-013-0367-2

Nagin, D. (1999). Analyzing developmental trajectories: A semiparametric, group-based approach. *PSYCHOLOGICAL METHODS*, *4*(2), 139–157. https://doi.org/10.1037/1082-989X.4.2.139

Pencina, M. J., D’Agostino, R. B. S., D’Agostino, R. B. J., & Vasan, R. S. (2008). Evaluating the added predictive ability of a new marker: From area under the ROC curve to reclassification and beyond. *Statistics in Medicine*, *27*(2), 157–172; discussion 207-212. https://doi.org/10.1002/sim.2929

Shimada, H., Doi, T., Lee, S., Makizako, H., Chen, L., & Arai, H. (2018). Cognitive Frailty Predicts Incident Dementia among Community-Dwelling Older People. *JOURNAL OF CLINICAL MEDICINE*, *7*(9). https://doi.org/10.3390/jcm7090250

**
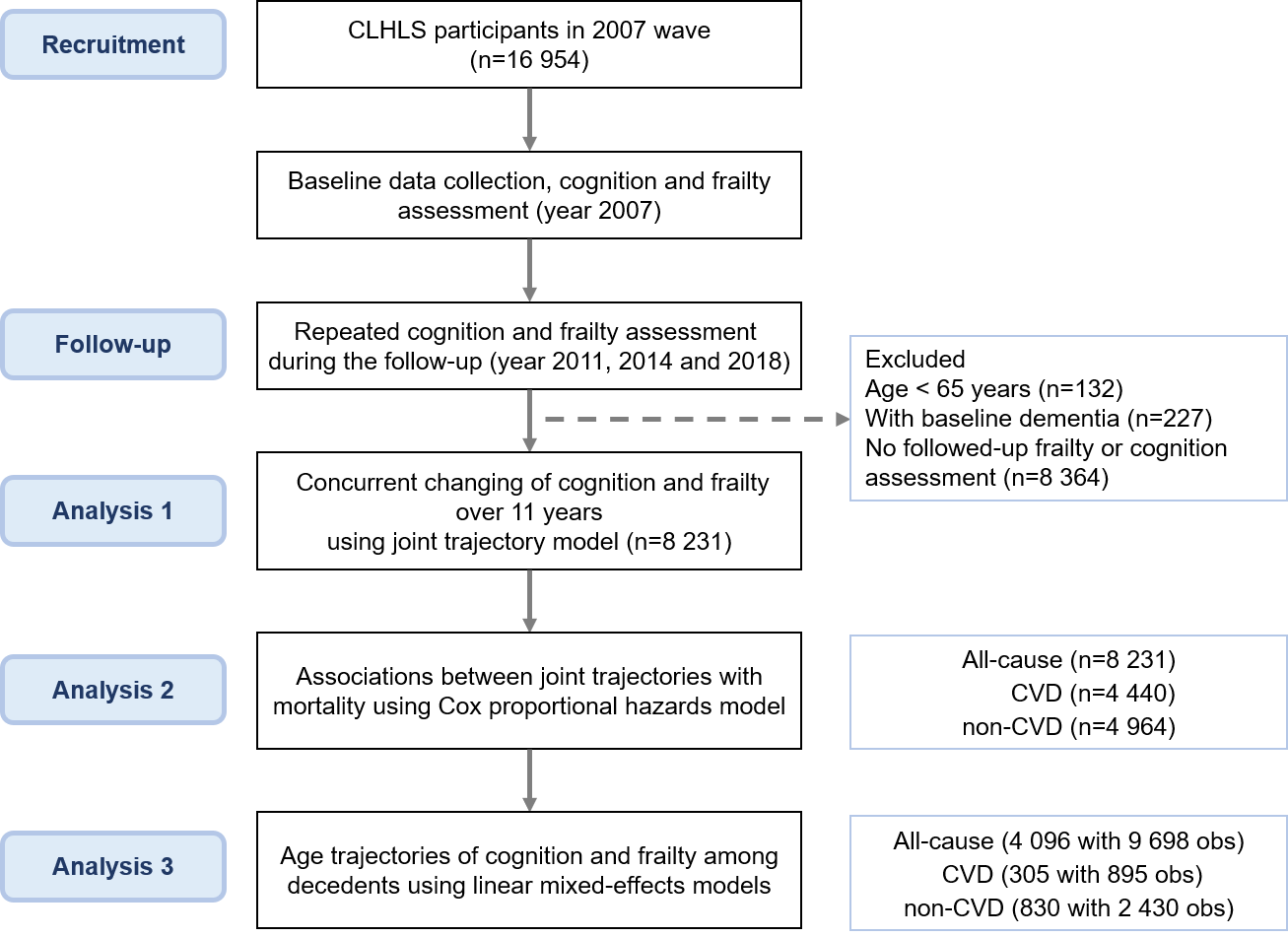
Figure S1. Flow chart of participants enrollment and analyses procedures of this study**

**
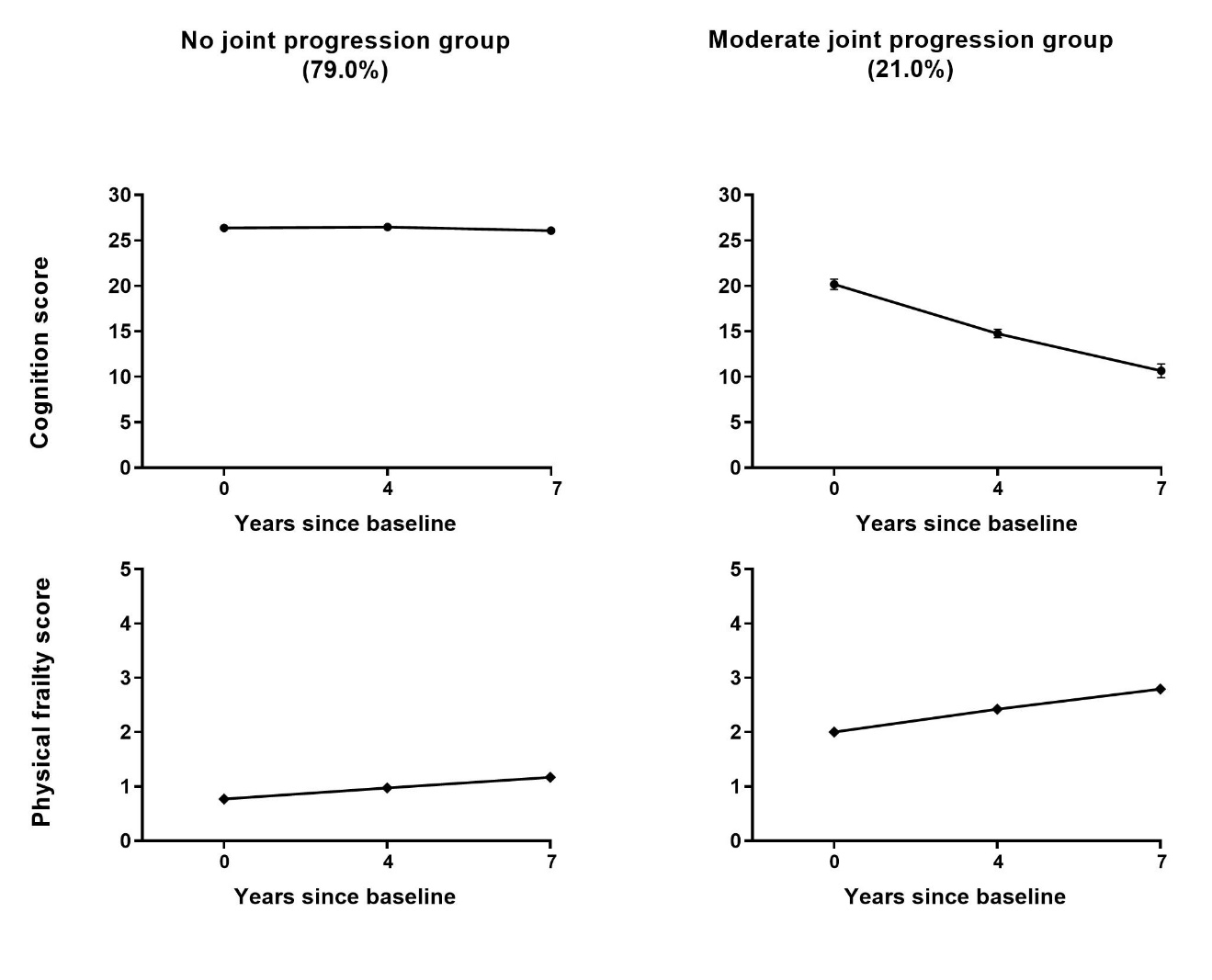
****Figure S2. Fitted joint trajectories among the CLHLS older adults with both three assessments of cognition and physical frailty (year 2007 – 2014, N=4,966).**

Cognition was assessed using the Mini-Mental State Examination (MMSE, score range: 0-30). Higher score indicates better cognition. Physical frailty was defined by the self-reported FRAIL phenotype with 5 components (fatigue, resistance, ambulation, illness, and weight loss). A higher score indicates worse frailty. ●, ◆ and the error bars represent the predicted values and 95% confidence intervals for MMSE score and physical frailty score, respectively. Two joint trajectories of cognition (top panel) and frailty (bottom panel) were identified as no joint progression (n = 3,925) and moderate joint progression (n = 1,041).

**Table S1. Posterior Probability of Assignment (PPA) of the 3 groups according to Joint Trajectory Model**

| **Assigned Trajectory Group**  **Based on the Maximal PPA^a^** | **Average PPA According to**  **the 3 Fitted Joint Trajectories** | | |
| --- | --- | --- | --- |
|  | No joint progression | Moderate joint progression | Rapid joint progression |
| No joint progression | **0.91^b^** | 0.09 | 0.00 |
| Moderate joint progression | 0.04 | **0.88^b^** | 0.08 |
| Rapid joint progression | 0.00 | 0.11 | **0.89^b^** |

*Notes:* ^a^ Based on the maximal PPA, a person was assigned to different Joint Trajectories.

^b^ Bolded values represent the average PPA for those with maximal PPA for a specific joint trajectory group.

**Table S2. Estimated joint trajectories and group-specific growth parameters**

| **Parameters estimates** | **No joint progression** | **Moderate joint progression** | **Rapid joint progression** |
| --- | --- | --- | --- |
| Predicted group probability (%) | 34.38 | 47.01 | 18.61 |
| Growth parameter | | | |
| Cognition (MMSE score). censored normal model | | | |
| Intercept | 30.20^**^ | 25.36^**^ | 13.11^**^ |
| Linear term | -0.13^**^ | -0.55^**^ | -2.33^**^ |
| Quadratic term | - | - | 0.08^*^ |
| Frailty (Fried phenotype). Zero-inflated Poisson model | | | |
| Intercept | -1.11^**^ | 0.50^**^ | 0.84^**^ |
| Linear term | 0.14^**^ | 0.04^**^ | 0.08^**^ |
| Quadratic term | - | - | -0.01^**^ |
| Model fit statistics |  |  |  |
| Bayesian information criterion | -10600.3 |  |  |

*Notes:* MMSE, Mini-Mental State Examination.

^*^ p <.01, ^**^ p <.001.

**Table S3. Hazard ratios (95% CI) for all-cause, CVD, and non-CVD mortality according to the fitted joint trajectories of cognition and physical frailty for participants enrolled between 2007 and 2018**

| **Trajectory groups / HR** | | **All-cause mortality** | **CVD mortality** | **Non-CVD mortality** |
| --- | --- | --- | --- | --- |
| **Model 1** | | | | |
|  | No. of death / No. of participants | 4,096/8,231 | 305/4,440 | 830/4,964 |
|  | No joint progression | Ref. | Ref. | Ref. |
|  | Moderate joint progression | 2.10 (1.91-2.31) | 1.66 (1.24-2.22) | 1.95 (1.62-2.34) |
|  | Rapid joint progression | 3.74 (3.32-4.20) | 3.32 (2.19-5.04) | 3.40 (2.63-4.39) |
| **Model 2** | | | | |
|  | No. of death / No. of participants | 3,915/7,901 | 292/4,278 | 790/4,775 |
|  | No joint progression | Ref. | Ref. | Ref. |
|  | Moderate joint progression | 2.06 (1.87-2.27) | 1.66 (1.23-2.24) | 1.94 (1.61-2.34) |
|  | Rapid joint progression | 3.58 (3.17-4.03) | 3.44 (2.24-5.27) | 3.14 (2.41-4.10) |
| **Model 3** | | | | |
|  | No. of death / No. of participants | 3,904/7,884 | 292/4,272 | 786/4,765 |
|  | No joint progression | Ref. | Ref. | Ref. |
|  | Moderate joint progression | 1.97 (1.79-2.17) | 1.58 (1.16-2.14) | 1.86 (1.54-2.25) |
|  | Rapid joint progression | 3.37 (2.99-3.81) | 3.21 (2.08-4.96) | 2.99 (2.28-3.92) |

*Notes:* HR, hazard ratio; CI, confidence interval.

Model 1 was adjusted for age, sex, education, baseline cognition and baseline frailty scores.

Model 2 was additionally adjusted for smoking, drinking, exercise, adequate medical service, SBP, DBP, and heart rate based on model 1.

Model 3 was additionally adjusted for ethnicity, residence, living arrangement, marital status, income levels, economic independence, and occupational status based on model 2.

**Table S4. Cumulative incidences for all-cause death, CVD death, and non-CVD death in the Chinese older adults across 11 years of follow-up according to the fitted joint trajectories of cognition and physical frailty [Wave 2007-2018]**

|  | **All-cause death** | **CVD death** | **Non-CVD death** |
| --- | --- | --- | --- |
| **No. of participants** | 8,231 | 4,440 | 4,964 |
| **4 Years Cumulative Incidence (95% CI)** |  |  |  |
| No joint progression | 2.6 (2.0-3.2%) | 0.0 (0.0-0.0%) | 0.0 (0.0-0.0%) |
| Moderate joint progression | 10.2 (9.2-11.1%) | 0.0 (0.0-0.0%) | 0.2 (0.0-0.4%) |
| Rapid joint progression | 25.1 (22.9-27.2%) | 0.3 (0.0-0.9%) | 1.4 (0.3-2.4%) |
| **7 Years Cumulative Incidence (95% CI)** |  |  |  |
| No joint progression | 14.4 (13.0-15.6%) | 1.1 (0.7-1.6%) | 2.3 (1.7-2.9%) |
| Moderate joint progression | 40.9 (39.3-42.5%) | 2.5 (1.7-3.2%) | 8.3 (7.1-9.5%) |
| Rapid joint progression | 73.1 (70.7-75.3%) | 11.9 (8.0-15.7%) | 23.9 (19.4-28.1%) |
| **11 Years Cumulative Incidence (95% CI)** |  |  |  |
| No joint progression | 32.2 (28.7-35.5%) | 6.0 (4.4-7.6%) | 14.0 (10.8-17.0%) |
| Moderate joint progression | 66.1 (63.3-68.7%) | 12.7 (10.1-15.1%) | 31.6 (27.6-35.4%) |
| Rapid joint progression | 93.6 (88.5-96.4%) | 27.9 (20.0-35.1%) | 54.9 (47.0-61.7%) |

*Notes:* CI, confidence interval; CVD, cardiovascular disease

**Table S5. Baseline characteristics of the included vs excluded participants (N=16954) [Wave 2007-2018]**

| **Characteristics** | **Total**  **(N=16954)** | **Excluded**  **(N=8723)** | **Included**  **(N=8231)** | ***P* values** |
| --- | --- | --- | --- | --- |
| Age, mean ± SD, years | 86.8 ± 12.0 | 90.6 ± 11.8 | 82.9 ± 10.8 | <0.0001 |
| Sex, women (%) | 9702 (57.2) | 5184 (59.4) | 4518 (54.9) | <0.0001 |
| Han nationality, yes (%) | 15918 (93.9) | 8193 (93.9) | 7725 (93.9) | 0.8458 |
| Rural residence, yes (%) | 10293 (60.7) | 5205 (59.7) | 5088 (61.8) | 0.0043 |
| Living alone, yes (%) | 2562 (15.1) | 1209 (13.9) | 1353 (16.4) | <0.0001 |
| More than 1 year of education, yes (%) ^a^ | 6371 (37.7) | 2862 (32.9) | 3509 (42.7) | <0.0001 |
| Currently married, yes (%) | 5251 (31.0) | 1899 (21.8) | 3352 (40.7) | <0.0001 |
| Income levels, richer, yes (%) ^a^ | 2250 (13.3) | 1125 (12.9) | 1125 (13.7) | 0.1504 |
| Economic independence, yes (%) | 4395 (25.9) | 1851 (21.2) | 2544 (30.9) | <0.0001 |
| Current smoking, yes (%) | 2966 (17.5) | 1307 (15.0) | 1659 (20.2) | <0.0001 |
| Current alcohol drinking, yes (%) | 2933 (17.3) | 1325 (15.2) | 1608 (19.5) | <0.0001 |
| Regular exercise, yes (%) ^a^ | 4645 (27.4) | 1920 (22.0) | 2725 (33.1) | <0.0001 |
| Adequate medical service, yes (%) | 15667 (92.4) | 7986 (91.6) | 7681 (93.3) | <0.0001 |
| Occupation, governmental, yes (%) ^a^ | 1183 (7.0) | 539 (6.2) | 644 (7.8) | <0.0001 |
| SBP, mean ± SD, mmHg ^a^ | 133.4 ± 24.2 | 132.3 ± 24.0 | 134.6 ± 24.3 | <0.0001 |
| DBP, mean ± SD, mmHg ^a^ | 77.7 ± 13.6 | 78.0 ± 13.7 | 77.5 ± 13.4 | 0.0246 |
| Heart Rate, mean ± SD, bpm ^a^ | 72.5 ± 11.6 | 72.6 ± 11.6 | 72.3 ± 11.6 | 0.0045 |
| Frailty score, mean ± SD | 1.6 ± 1.2 | 1.8 ± 1.1 | 1.3 ± 1.2 | <0.0001 |
| Frailty, yes (%) | 4781 (28.2) | 3143 (36.0) | 1638 (19.9) | <0.0001 |
| MMSE score, mean ± SD | 20.7 ± 10.2 | 17.8 ± 11.0 | 23.7 ± 8.2 | <0.0001 |
| Cognitive impairment, yes (%) ^a^ | 5445 (32.2) | 3849 (44.3) | 1596 (19.4) | <0.0001 |

*Notes:* SBP, systolic blood pressure; DBP, diastolic blood pressure.

^a^ Of the 16954 older adults, numbers of missing data ranged from 1 to 875 (50 for education, 47 for income levels, 1 for exercise, 22 for occupational status, 872 for SBP and DBP, 875 for Heart Rate, 50 for cognitive groups).

**Table S6. Prediction performance on mortality among baseline cognition and / or frailty predictors and the joint trajectories using CLHLS data [Wave 2007-2018]**

|  | **Model Performance** ^a^ | | |
| --- | --- | --- | --- |
| **Model (based on model 3)**^b^ | **C-Statistic (95% CI)** | **IDI (95% CI)** | **NRI (95% CI)** |
| **All-cause mortality** |  |  |  |
| +Joint Trajectories ^c^ | 0.7986 (0.7888, 0.8083) | 0.0235 (0.0200, 0.0269)^***^ | 0.4015 (0.3583, 0.4447)^***^ |
| +Baseline frailty score | 0.7850 (0.7750, 0.7951) | -0.0009 (-0.0022, 0.0004) | 0.1127 (0.0686, 0.1567)^***^ |
| +Baseline cognitive score | 0.7862 (0.7761, 0.7962) | -0.0002 (-0.0011, 0.0007) | 0.0777 (0.0336, 0.1218)^**^ |
| +Baseline cognitive frailty ^d^ | 0.7861 (0.7761, 0.7962) | - | - |
| **CVD mortality** |  |  |  |
| +Joint Trajectories | 0.7743 (0.7641, 0.7845) | 0.0072 (0.0061, 0.0082) ^***^ | 0.4279 (0.3848, 0.471)^***^ |
| +Baseline frailty score | 0.7570 (0.7465, 0.7676) | -0.0016 (-0.0020, -0.0013)^***^ | 0.1563 (0.1123, 0.2003)^***^ |
| +Baseline cognitive score | 0.7556 (0.7450, 0.7662) | -0.0020 (-0.0025, -0.0016)^***^ | -0.0542 (-0.0975, -0.0109) |
| +Baseline cognitive frailty | 0.7581 (0.7476, 0.7687) | - | - |
| **Non-CVD mortality** |  |  |  |
| +Joint Trajectories | 0.7897 (0.7798, 0.7996) | 0.0145 (0.0127, 0.0164) ^***^ | 0.4124 (0.3692, 0.4556)^***^ |
| +Baseline frailty score | 0.7734 (0.7631, 0.7837) | -0.0029 (-0.0035, -0.0023)^***^ | -0.1088 (-0.1524, -0.0652)^***^ |
| +Baseline cognitive score | 0.7757 (0.7654, 0.7859) | -0.0001 (-0.0004, 0.0003) | 0.0280 (-0.0162, 0.0721) |
| +Baseline cognitive frailty | 0.7757 (0.7655, 0.7859) | - | - |

*Notes:* CI, confidence interval; IDI, integrated discrimination improvement; NRI, net reclassification index.

^*^p<.01; ^**^p<.001, ^***^p<.0001.

^a^ C-statistics were calculated in fully adjusted model 3 with four predictors showed in rows, respectively; NRI and IDI were calculated the added predictive values in models using the joint trajectories of cognition and frailty, baseline cognitive score and baseline frailty score in comparison to that of the models using baseline cognitive frailty.

^b^ Model 3 adjusted for age, sex, education, smoking, drinking, exercise, adequate medical service, SBP, DBP, heart rate, ethnicity, residence, living arrangement, marital status, income levels, economic independence, and occupational status.

^c^ Three joint trajectories of cognition and frailty were identified: *no joint progression* (n = 2,830), *moderate joint progression* (n = 3,869) and *rapid joint progression* (n = 1,532).

^d^ Baseline cognitive frailty was defined as the simultaneous presence of both cognitive impairment (considering MMSE score based on educational background) and physical frailty at baseline.

**Table S7. Hazard ratios (95% CI) for all-cause, CVD, and non-CVD mortality according to the fitted joint trajectories of cognition and physical frailty [Wave 2005-2018]**

| **Trajectory groups / HR (95% CI)** | | **All-cause mortality** | **CVD mortality** | **Non-CVD mortality** |
| --- | --- | --- | --- | --- |
| **Model 1** | | | | |
|  | No. of death / No. of participants | 4,198/7,452 | 202/3,456 | 745/3,999 |
|  | No joint progression | Ref. | Ref. | Ref. |
|  | Moderate joint progression | 2.02 (1.85-2.20) | 1.95 (1.36-2.78) | 2.25 (1.85-2.73) |
|  | Rapid joint progression | 3.19 (2.81-3.62) | 1.57 (0.69-3.57) | 3.31 (2.40-4.56) |
| **Model 2** | | | | |
|  | No. of death / No. of participants | 4,122/7,316 | 201/3,395 | 734/3,928 |
|  | No joint progression | Ref. | Ref. | Ref. |
|  | Moderate joint progression | 2.00 (1.83-2.19) | 2.02 (1.41-2.89) | 2.25 (1.85-2.75) |
|  | Rapid joint progression | 3.20 (2.81-3.63) | 1.62 (0.71-3.70) | 3.24 (2.35-4.46) |
| **Model 3** | | | | |
|  | No. of death / No. of participants | 4,096/7,278 | 201/3,383 | 729/3,911 |
|  | No joint progression | Ref. | Ref. | Ref. |
|  | Moderate joint progression | 1.92 (1.75-2.09) | 1.94 (1.35-2.77) | 2.16 (1.77-2.63) |
|  | Rapid joint progression | 3.11 (2.74-3.54) | 1.59 (0.70-3.61) | 3.23 (2.34-4.44) |

*Notes:* HR, hazard ratio; CI, confidence interval.

Model 1 was adjusted for age, sex, education, baseline cognition and baseline frailty scores.

Model 2 was additionally adjusted for smoking, drinking, exercise, adequate medical service, SBP, DBP, and heart rate based on model 1.

Model 3 was additionally adjusted for ethnicity, residence, living arrangement, marital status, income levels, economic independence, and occupational status based on model 2.

**Table S8. Prediction performance on mortality among baseline cognition and frailty predictors and the joint trajectories [Wave 2005-2018]**

|  | | **Model Performance** ^a^ | | | |
| --- | --- | --- | --- | --- | --- |
| **Model (based on model 3)**^b^ | **C-Statistic (95% CI)** | | **IDI (95% CI)** | **NRI (95% CI)** |  |
| **All-cause mortality** |  | |  |  |  |
| +Joint Trajectories ^c^ | 0.7841 (0.7736, 0.7946) | | 0.0237 (0.0202, 0.0272)^***^ | 0.4057 (0.3611, 0.4502)^***^ |  |
| +Baseline frailty score | 0.7702 (0.7594, 0.7810) | | 0.0000 (-0.0001, 0.0001) | -0.1129 (-0.1588, -0.0670)^***^ |  |
| +Baseline cognitive score | 0.7702 (0.7594, 0.7810) | | 0.0000 (-0.0001, 0.0001) | -0.1129 (-0.1588, -0.0670)^***^ |  |
| +Baseline cognitive frailty ^d^ | 0.7702 (0.7594, 0.7810) | | - | - |  |
| **CVD mortality** |  | |  |  |  |
| +Joint Trajectories | 0.7011 (0.6892, 0.7130) | | 0.0018 (0.0014, 0.0021)^***^ | 0.2117 (0.1660, 0.2574)^***^ |  |
| +Baseline frailty score | 0.6911 (0.6790, 0.7031) | | 0.0000 (-0.0003,0.0002) | -0.0808 (-0.1267, -0.0349)^**^ |  |
| +Baseline cognitive score | 0.6911 (0.6790, 0.7031) | | 0.0000 (-0.0003,0.0002) | -0.0808 (-0.1267, -0.0349) ^**^ |  |
| +Baseline cognitive frailty | 0.6882 (0.6761, 0.7003) | | - | - |  |
| **Non-CVD mortality** |  | |  |  |  |
| +Joint Trajectories | 0.7897 (0.7798, 0.7996) | | 0.0145 (0.0127, 0.0164)^***^ | 0.4124 (0.3692, 0.4556)^***^ |  |
| +Baseline frailty score | 0.7734 (0.7631, 0.7837) | | -0.0029 (-0.0035, -0.0023)^***^ | -0.1088 (-0.1524, -0.0652)^***^ |  |
| +Baseline cognitive score | 0.7757 (0.7654, 0.7859) | | -0.0001 (-0.0004, 0.0003) | 0.0280 (-0.0162, 0.0721) |  |
| +Baseline cognitive frailty | 0.7757 (0.7655, 0.7859) | | - | - |  |

*Notes:* CI, confidence interval; IDI, integrated discrimination improvement; NRI, net reclassification index.

^*^p<.01; ^**^p<.001, ^***^p<.0001.

^a^ C-statistics were calculated in fully adjusted model 3 with four predictors showed in rows, respectively; NRI and IDI were calculated the added predictive values in models using the joint trajectories of cognition and frailty, baseline cognitive score and baseline frailty score in comparison to that of the models using baseline cognitive frailty.

^b^ Model 3 was adjusted for age, sex, education, baseline cognition, baseline frailty, smoking, drinking, exercise, adequate medical service, SBP, DBP, heart rate, ethnicity, residence, living arrangement, marital status, income levels, economic independence, and occupational status.

^c^ Three joint trajectories of cognition and frailty were identified: *no joint progression* (n = 3,113), *moderate joint progression* (n = 3,197) and *rapid joint progression* (n = 1,142).

^d^ Baseline cognitive frailty was defined as the simultaneous presence of both cognitive impairment (considering MMSE score based on educational background) and physical frailty at baseline.

**Table S9. Posterior Probability of Assignment (PPA) of the 2 groups according to Joint Trajectory Model among older adults who had both three assessments of cognition and physical frailty (Wave 2007 – 2014, N=4,966)**

| **Assigned Trajectory Group**  **Based on the Maximal PPA^a^** | **Average PPA According to**  **the 2 Fitted Joint Trajectories** | |
| --- | --- | --- |
|  | No joint progression | Moderate joint progression |
| No joint progression | **0.98^b^** | 0.02 |
| Moderate joint progression | 0.06 | **0.94^b^** |

*Notes:* ^a^ Based on the maximal PPA, a person was assigned to different joint trajectories.

^b^ Bolded values represent the average PPA for those with maximal PPA for a specific joint trajectory group.

**Table S10. Estimated joint trajectories and group-specific growth parameters among older adults who had both three assessments of cognition and physical frailty (Wave 2007 – 2014, N=4,966)**

| **Parameters estimates** | No joint progression | Moderate joint progression |
| --- | --- | --- |
| Predicted group probability (%) | 79.04 | 20.96 |
| Growth parameter | | |
| Cognition (MMSE score). censored normal model | | |
| Intercept | 28.219^***^ | 20.388^***^ |
| Linear term | 0.198^*^ | -1.413^***^ |
| Quadratic term | -0.039^**^ | - |
| Frailty (Fried phenotype). Zero-inflated Poisson model | | |
| Intercept | -0.265^***^ | 0.695^***^ |
| Linear term | 0.060^***^ | 0.047^***^ |
| Model fit statistics |  |  |
| Bayesian information criterion | -64073.47 |  |

*Notes:* MMSE, Mini-Mental State Examination.

^*^ p <.05, ^**^ p <.01, ^***^ p <.0001.

**Table S11. Hazard ratios (95% CI) for all-cause, CVD, and non-CVD mortality according to the fitted joint trajectories among older adults who had both three assessments of cognition and physical frailty (Wave 2007 – 2014, N=4,966)**

| **Trajectory groups / HR** | **All-cause mortality** | **CVD mortality** | **Non-CVD mortality** |
| --- | --- | --- | --- |
| **Model 1** | | | |
| No. of death / No. of participants | 1,483/4,966 | 281/3,764 | 759/4,241 |
| No joint progression | Ref. | Ref. | Ref. |
| Moderate joint progression | 2.51 (2.21-2.85) | 2.66 (1.97-3.59) | 2.40 (2.00-2.88) |
| **Model 2** |  |  |  |
| No. of death / No. of participants | 1,423/4,796 | 269/3,642 | 725/4,097 |
| No joint progression | Ref. | Ref. | Ref. |
| Moderate joint progression | 2.46 (2.15-2.81) | 2.77 (2.04-3.78) | 2.31 (1.91-2.79) |
| **Model 3** | | | |
| No. of death / No. of participants | 1,419/4,786 | 269/3,636 | 722/4,088 |
| No joint progression | Ref. | Ref. | Ref. |
| Moderate joint progression | 2.43 (2.13-2.78) | 2.79 (2.04-3.80) | 2.29 (1.89-2.77) |

*Notes:* HR, hazard ratio; CI, confidence interval.

Model 1 was adjusted for age, sex, education, baseline cognition and baseline frailty scores.

Model 2 was additionally adjusted for smoking, drinking, exercise, adequate medical service, SBP, DBP, and heart rate based on model 1.

Model 3 was additionally adjusted for ethnicity, residence, living arrangement, marital status, income levels, economic independence, and occupational status based on model 2.
